# Interfacial Microchemistry and Surface Stability of Monolithic and Layered Dental Restorative Systems: A Comparative SEM–EDS–XPS Study

**DOI:** 10.3390/dj14090596

**Published:** 2026-09-15

**Authors:** Cristian Boanca, Dorin Ioan Cocoș, Ramona Feier, Kamel Earar

**Affiliations:** 1 Medical and Pharmaceutical Research Center, Faculty of Medicine and Pharmacy, “Dunărea de Jos” University of Galati, 800008 Galati, Romania; andiboanca@yahoo.com (C.B.); kamel.earar@ugal.ro (K.E.); 2 Dental-Medicine Department, Faculty of Medicine and Pharmacy, “Dunărea de Jos” University of Galati, 800201 Galati, Romania; 3 Department of Dental Medicine, Dimitrie Cantemir University, 540545 Târgu Mureș, Romania

**Keywords:** dental ceramics, zirconia, cobalt–chromium alloy, feldspathic ceramic, metal–ceramic interface, SEM–EDS, XPS, surface chemistry

## Abstract

**Background/Objectives:** Restorative system configuration may influence the surface chemistry and interfacial behavior of dental ceramic and metal–ceramic systems, but direct comparative evaluations integrating SEM, EDS, and XPS remain limited. **Methods:** This in vitro study compared three restorative configurations: Monolithic ZrO_2_, Feldspar/ZrO_2_, and Feldspar/Co-Cr. Thirty specimens were fabricated and allocated into three groups (*n* = 10/group). Surface and cross-sectional morphology were assessed by scanning electron microscopy, while elemental composition and interfacial transition patterns were evaluated using energy-dispersive X-ray spectroscopy, including point analysis, elemental mapping, and line-scan profiles. X-ray photoelectron spectroscopy was used to characterize material-specific surface chemical profiles. **Results:** Monolithic ZrO_2_ showed a compact and homogeneous ceramic morphology, with a dominant Zr–O–Y chemical profile. Feldspar/ZrO_2_ specimens exhibited a sharply defined ceramic–ceramic interface, with limited elemental overlap between the feldspathic veneer and zirconia core. In contrast, Feldspar/Co-Cr specimens showed a broader ceramic–metal transition zone, mainly supported by SEM–EDS evidence of Cr-, Co-, and O-related interfacial signals. XPS provided complementary surface chemical information, without definitive oxide phase identification. **Conclusions:** Within the limitations of this in vitro study, the analyzed restorative configurations exhibited distinct morphological, elemental, and surface chemical patterns. Feldspar/Co-Cr showed a broader ceramic–metal elemental transition than Feldspar/ZrO_2_; however, the present findings should not be interpreted as evidence of superior bond strength, mechanical stability, fatigue resistance, or clinical performance. Further mechanical and aging studies are required to determine the functional significance of these interfacial differences.

## 1. Introduction

The continuous development of dental ceramics and ceramic-based restorative materials has changed the design, fabrication, and clinical selection of fixed prosthetic restorations. Among these materials, yttria-stabilized zirconia has become widely used because of its favorable mechanical properties, chemical stability, digital processability, and broad clinical applicability [1,2]. The transition from conventionally veneered restorations toward monolithic and digitally manufactured systems has reduced some veneer-related complications, while increasing the need to understand how material architecture, processing route, surface chemistry, and interfacial behavior influence restorative performance [1,2,3].

Monolithic zirconia restorations are clinically attractive because they eliminate the veneer–core interface, a structurally sensitive region in layered ceramic systems [1,2]. However, zirconia surface behavior remains influenced by chemical composition, yttria stabilization, phase stability, aging, surface treatment, and interaction with luting or veneering materials [4,5,6,7]. Surface conditioning and etching strategies may modify zirconia topography and bonding potential [4,5,8], while aging-related changes and yttrium-related stabilization mechanisms may affect long-term surface stability [6,7]. Therefore, zirconia requires not only morphological assessment, but also elemental and surface chemical characterization [6,7,8].

Despite the increased use of monolithic zirconia, bilayered zirconia-based restorations remain clinically relevant when improved esthetics are required [1,2,9,10,11,12,13]. In these systems, feldspathic veneering ceramic is applied over a zirconia core, creating a ceramic–ceramic interface whose integrity is essential for restoration performance [10,11,12,13]. This interface is influenced by surface treatment, veneering technique, liner application, ceramic composition, firing protocol, and interfacial microstructure. Because limited chemical interaction, mechanical adaptation, and residual stress distribution may contribute to chipping or delamination, detailed microstructural and microchemical assessment remains important [13,14,15].

Cobalt–chromium metal–ceramic restorations also remain clinically relevant because of their mechanical reliability, cost-effectiveness, and established use in fixed prosthodontics [16,17]. In contrast to ceramic–ceramic systems, the metal–ceramic interface is influenced by alloy composition, surface preparation, oxidation behavior, fabrication technique, and oxide-associated interfacial regions involved in ceramic bonding [18,19,20,21]. Modern manufacturing techniques, including selective laser melting, as well as thermal cycling and surface treatments, may further affect the microstructure, surface roughness, chemical composition, ceramic bond strength, and interfacial stability of Co-Cr frameworks [20,21,22,23,24].

Although Monolithic ZrO_2_, Feldspar/ZrO_2_, and Feldspar/Co-Cr systems are all clinically relevant, they are governed by different stabilization and bonding mechanisms. Monolithic zirconia relies mainly on intrinsic ceramic compactness, phase stability, and Zr–O–Y surface chemistry [1,4,6,7,8]; Feldspar/ZrO_2_ systems depend on ceramic–ceramic adaptation and zirconia–ceramic interfacial integrity [13,14,15]; and Feldspar/Co-Cr systems involve a ceramic–metal interface influenced by alloy composition, manufacturing route, surface preparation, and oxide-associated chemical contributions [19,20,21]. However, direct comparative evaluations integrating scanning electron microscopy, energy-dispersive X-ray spectroscopy, and X-ray photoelectron spectroscopy across these three restorative architectures remain limited.

Therefore, this study aimed to comparatively evaluate the surface morphology, elemental composition, surface chemical signatures, and interfacial transition behavior of Monolithic ZrO_2_, Feldspar/ZrO_2_, and Feldspar/Co-Cr restorative systems. The specific scientific advance of the present study is the direct comparison, under standardized experimental conditions, of architecture-dependent interfacial transition behavior across these restorative systems. In particular, the layered systems were evaluated for differences in the spatial extent and elemental overlap of the veneer–substrate transition zones using cross-sectional SEM and spatially resolved EDS line-scan/mapping analysis, while XPS provided complementary surface chemical information. This integrated approach was intended to determine whether ceramic–ceramic and ceramic–metal restorative architectures exhibit measurably distinct interfacial microchemical organization beyond their expected constituent elemental profiles. The null hypothesis was that no significant differences would be observed among the three systems in terms of morphology, elemental distribution, surface chemical signatures, or interfacial transition characteristics.

## 2. Materials and Methods

### 2.1. Study Design, Materials, and Experimental Groups

This in vitro study was designed as a comparative microstructural and microchemical investigation of three clinically relevant dental restorative systems, focusing on the relationship between restorative system configuration, surface chemistry, and interfacial behavior. A total of 30 independently fabricated specimens were allocated to three experimental groups according to material configuration, with 10 specimens per group (*n* = 10/group). The sample size of 10 independent specimens per group was selected to provide specimen-level replication appropriate for the exploratory comparative SEM–EDS–XPS design. No formal prospective power calculation was performed before specimen fabrication. A sensitivity analysis based on *n* = 10 specimens per group, α = 0.05, and 80% statistical power indicated that the study was primarily capable of detecting large between-group effects, corresponding approximately to Cohen’s d = 1.32 for two-group comparisons and Cohen’s f = 0.60 for three-group comparisons. Accordingly, the study was not designed to reliably detect small or moderate between-group effects.

Group 1, Monolithic ZrO_2_, consisted of yttria-stabilized tetragonal zirconia polycrystal specimens fabricated from IPS e.max ZirCAD blocks (Ivoclar Vivadent, Schaan, Liechtenstein).

Group 2, Feldspar/ZrO_2_, consisted of zirconia cores fabricated from the same Y-TZP material and veneered with fluorapatite feldspathic ceramic (IPS e.max Ceram, Ivoclar Vivadent, Schaan, Liechtenstein).

Group 3, Feldspar/Co-Cr, consisted of cobalt–chromium alloy frameworks (Colado CC, Ivoclar Vivadent, Schaan, Liechtenstein) veneered with leucite-containing feldspathic ceramic (IPS InLine, Ivoclar Vivadent, Schaan, Liechtenstein). These groups represented three clinically relevant restorative system configurations used in fixed prosthodontics: monolithic ceramic, ceramic–ceramic layered, and metal–ceramic layered systems.

The experimental groups were designed to represent clinically relevant restorative system configurations rather than to isolate restorative architecture as an independent variable. Accordingly, differences among groups inherently included variations in substrate composition, veneering ceramic, and fabrication workflow. The term “restorative architecture” is therefore used in this study to describe the overall material–structural configuration of each system, rather than a single experimentally isolated factor.

All specimens were fabricated under standardized laboratory conditions as plate-shaped samples with comparable external dimensions. Monolithic ZrO_2_ specimens had a target final thickness of 1.0–1.5 mm. In the Feldspar/ZrO_2_ group, the zirconia core was fabricated with a nominal thickness of approximately 0.5 mm, while the fluorapatite feldspathic veneering ceramic was applied to a thickness of approximately 0.7–1.0 mm, resulting in a final specimen thickness of approximately 1.2–1.5 mm. In the Feldspar/Co-Cr group, the Co-Cr framework had a nominal thickness of approximately 0.4–0.5 mm, and the leucite-containing feldspathic veneering ceramic was applied to a thickness of approximately 0.7–1.0 mm, resulting in a final specimen thickness of approximately 1.1–1.5 mm. The individual layer dimensions were maintained consistently within each experimental group to obtain comparable cross-sectional architecture and to minimize geometry-related variability in the subsequent interfacial analyses. Only specimens with intact morphology, preserved marginal integrity, and no visible porosity, cracks, warpage, contamination, incomplete firing, marginal chipping, veneer detachment, or delamination were included. The null hypothesis was that no significant differences would be observed among the three systems in terms of surface morphology, elemental composition, surface chemical signatures, or interfacial transition characteristics.

### 2.2. Specimen Fabrication Protocol

Monolithic ZrO_2_ specimens and zirconia cores for the Feldspar/ZrO_2_ group were milled using a CAD/CAM milling unit (PrograMill PM7, Ivoclar Vivadent, Schaan, Liechtenstein). The milled zirconia specimens were sintered in a zirconia furnace (Programat S1 1600, Ivoclar Vivadent, Schaan, Liechtenstein) using the manufacturer-recommended sintering program for the selected zirconia material. For the Feldspar/ZrO_2_ group, the sintered zirconia cores were veneered using a standardized layering technique with IPS e.max Ceram and fired in a ceramic furnace (Programat P710, Ivoclar Vivadent, Schaan, Liechtenstein) according to the manufacturer-recommended firing protocol.

For the Feldspar/Co-Cr group, cobalt–chromium frameworks were fabricated by selective laser melting (EOSINT M 270, EOS GmbH, Krailling, Germany) using validated laboratory parameters recommended for the selected Co-Cr alloy system. Before veneering, the Co-Cr frameworks were finished, ultrasonically cleaned, and subjected to a manufacturer-recommended oxidation firing cycle to promote oxide-layer formation before feldspathic ceramic application. The feldspathic ceramic was then applied using the same standardized layering approach with IPS InLine and fired in the Programat P710 furnace according to the manufacturer-recommended firing protocol.

After fabrication, all specimens were visually inspected for defects, ultrasonically cleaned in distilled water for 10 min, rinsed with deionized water, dried with oil-free compressed air, and stored in closed dust-free containers at room temperature until SEM, EDS, and XPS analyses.

### 2.3. Surface and Cross-Sectional Preparation

Before analysis, all specimens were macroscopically inspected to confirm surface integrity and the absence of visible defects, including cracks, chipping, porosity, delamination, or contamination. For surface analysis, the external restorative surface was evaluated directly. For interfacial analysis, specimens from the layered groups were sectioned perpendicular to the veneer–substrate interface using a low-speed diamond saw under continuous water cooling to minimize heat generation and sectioning-induced damage.

The exposed cross-sections were progressively polished with silicon carbide abrasive papers of 600, 800, 1200, 2000, and 4000 grit, followed by final polishing with 1 µm diamond suspension. To minimize preparation-induced material transfer across the veneer–substrate interface, polishing was performed under low applied pressure, with thorough rinsing between successive abrasive steps. After final polishing, the specimens were ultrasonically cleaned in distilled water to remove residual abrasive particles and loosely adherent debris, rinsed with deionized water, and dried with oil-free compressed air. Before EDS acquisition, the prepared cross-sections were screened by SEM, and areas showing polishing scratches, particulate contamination, edge damage, or visible evidence of material dragging across the interface were excluded from analysis. EDS spectra, line scans, and elemental maps were acquired only from morphologically intact and contamination-free interfacial regions. The prepared cross-sections were then used to assess interfacial morphology and elemental transition patterns between the feldspathic veneer and the underlying zirconia or Co-Cr substrate.

### 2.4. Scanning Electron Microscopy Analysis

Scanning electron microscopy (SEM) was performed using a Quanta 200 microscope (FEI Company, Hillsboro, OR, USA) in low-vacuum mode under operating conditions adjusted according to the analytical purpose, with a working distance of 10 mm. SEM and EDS analyses were performed on uncoated specimens in low-vacuum mode to reduce charging artifacts while avoiding coating-related interference with elemental analysis. For spatially resolved interfacial EDS analysis, an accelerating voltage of 10 kV was used to limit the electron interaction volume and preserve micrometer-scale spatial resolution during line-scan and elemental mapping measurements. Regional compositional EDS point spectra were acquired separately at 20 kV, as described in Section 2.5. Representative surface and cross-sectional areas were examined at 500× and 2000× magnification, respectively, and scale bars were included in all representative micrographs. SEM analysis was used to evaluate surface morphology, structural continuity, and interfacial characteristics of the analyzed restorative systems, including homogeneity, compactness, porosity, microcracks, gap formation, delamination, and transition-zone morphology, as applicable.

In the layered groups, particular attention was given to the veneer–substrate interface. Cross-sectional SEM micrographs were used to evaluate interfacial morphology and to support localization of the transition zone, whereas quantitative interfacial transition width was determined according to the combined SEM–EDS line-scan protocol described in Section 2.7. Image-based measurements were performed using ImageJ v1.54.

SEM-derived morphometric and semi-quantitative parameters were assessed according to predefined criteria. For surface-based measurements, a standardized SEM field was defined as a surface micrograph acquired at 500× magnification under identical acquisition conditions across all experimental groups. Five non-overlapping surface fields were evaluated per specimen. Surface defect density was defined as the number of visible surface discontinuities, including pores, localized irregularities, and other clearly identifiable microstructural defects, recorded within each standardized field. Microcrack count was defined as the number of clearly identifiable linear discontinuities within the same standardized field. Visible porosity was scored for each field using a four-point scale: 0 = absent/negligible, 1 = low, 2 = moderate, and 3 = marked porosity. For each surface parameter, the five field-level observations were averaged to obtain a single specimen-level value, which was subsequently used for group-level statistical analysis. In the layered groups, interfacial continuity was assessed separately on cross-sectional SEM images acquired at 2000× magnification using a five-point scale: 1 = marked discontinuity or gap formation, 2 = partial discontinuity, 3 = moderate continuity with localized irregularities, 4 = good continuity with minor irregularities, and 5 = continuous interface without visible gaps or delamination.

All SEM-derived morphometric and semi-quantitative assessments were performed independently by two calibrated evaluators who were blinded to the experimental group assignment. Before formal analysis, both evaluators underwent a calibration session using a separate set of representative SEM images to standardize the assessment of surface defect density, visible porosity, microcrack count, and interfacial continuity according to the predefined criteria. Each evaluator subsequently assessed all coded images independently. To determine intra-examiner repeatability, 25% of the image set was randomly selected and reassessed independently by each evaluator after a 2-week interval under the same blinded conditions. Disagreements affecting the final classification were resolved by consensus only after completion of the independent assessments.

### 2.5. Energy-Dispersive X-Ray Spectroscopy Analysis

Energy-dispersive X-ray spectroscopy (EDS) was performed using an EDAX Genesis detector/software package (v6.29, EDAX Inc., Mahwah, NJ, USA) coupled to the Quanta 200 SEM system (FEI Company, Hillsboro, OR, USA). Spectra were acquired from representative surface, cross-sectional, and interfacial regions, as applicable, to determine the elemental composition of each restorative system. Regional EDS point spectra used for compositional characterization were acquired at an accelerating voltage of 20 kV, providing broader excitation of characteristic X-ray lines for elemental identification and regional compositional assessment. In contrast, interfacial EDS line scans and elemental maps were acquired at 10 kV. For interfacial line-scan acquisition, a step size of 0.20 µm and a probe setting of 3.0 were used, corresponding to an estimated lateral EDS spatial resolution of approximately 0.8–1.0 µm under the selected acquisition conditions. The lower accelerating voltage used for the interfacial analyses was deliberately selected to reduce the electron interaction volume and improve spatial resolution across the micrometer-scale veneer–substrate transition zones. Accordingly, regional point spectra were used for compositional characterization, whereas the 10 kV line-scan and mapping data were interpreted primarily as spatially resolved comparative microchemical profiles.

For monolithic ZrO_2_, EDS analysis focused on Zr, O, Y, and trace accessory elements. In the Feldspar/ZrO_2_ group, spectra were acquired from the feldspathic veneer, zirconia core, and interface, with attention to Si, Al, Na, K, O, Zr, and Y. In the Feldspar/Co-Cr group, spectra were obtained from the feldspathic veneer, Co-Cr substrate, and interface, focusing on Si, Al, Na, K, O, Co, Cr, and other alloy-associated elements. EDS-derived compositional values were reported as weight percentages (wt%), with minor heavy-element contributions interpreted semi-quantitatively under the selected acquisition conditions.

In the layered groups, EDS line scans and elemental maps were performed across the veneer–substrate interface. Line-scan trajectories were positioned perpendicular to the interface to evaluate elemental distribution, elemental overlap, and the abruptness or gradualness of the interfacial transition. Three independent line scans were acquired from non-overlapping interfacial locations in each layered specimen, as detailed in Section 2.7. The EDS profiles were interpreted together with SEM and XPS findings. Oxygen values obtained by EDS were interpreted semi-quantitatively because light-element quantification by EDS may be affected by matrix effects, surface topography, detector sensitivity, and acquisition conditions. Minor heavy-element contributions were likewise interpreted cautiously because their quantitative reliability may vary according to the selected characteristic lines, matrix effects, spectral overlap, and acquisition conditions.

### 2.6. X-Ray Photoelectron Spectroscopy Analysis

X-ray photoelectron spectroscopy (XPS) was performed using an ESCALAB 250Xi spectrometer (Thermo Fisher Scientific, Waltham, MA, USA) equipped with a monochromatic Al Kα X-ray source. Survey spectra were acquired for all experimental groups to identify the main surface elements and compare the chemical profiles of the analyzed restorative systems.

For monolithic ZrO_2_, XPS analysis focused on Zr-, O-, and Y-related signals. For Feldspar/ZrO_2_, feldspathic ceramic-related signals, including Si, O, Al, Na, and K, were evaluated together with possible weak zirconia-associated contributions. For Feldspar/Co-Cr, feldspathic ceramic-related signals were assessed together with possible Cr-, Co-, and O-associated features. These signals were interpreted as complementary surface chemical information and were not considered the primary evidence for defining the buried veneer–substrate interface.

Spectra were interpreted qualitatively and semi-quantitatively based on peak identification and relative signal intensity. Binding energy calibration was performed using the adventitious carbon C 1s peak at 284.8 eV, when required. Adventitious carbon was used solely as a binding-energy reference and was not assumed to be present at an identical surface concentration across specimens. Differences in C 1s atomic percentage were interpreted as variations in adventitious surface hydrocarbon coverage rather than as differences in bulk material composition. Because XPS is highly surface-sensitive, variations in hydrocarbon coverage may partially attenuate the relative intensities of underlying material-specific photoelectron signals, including Zr- and Si-related signals. Accordingly, XPS-derived atomic percentages were interpreted as comparative semi-quantitative surface chemical profiles rather than as direct measurements of bulk stoichiometric composition. In the layered groups, interfacial transition behavior was primarily assessed using cross-sectional SEM observations and EDS line-scan/mapping analysis, whereas XPS was used to complement the material-specific surface chemical profile. Because the analysis was based primarily on survey spectra, oxide-associated features were interpreted cautiously and were not considered definitive phase identification in the absence of high-resolution peak deconvolution. XPS findings were therefore interpreted in correlation with, but not as a substitute for, SEM and EDS interfacial evidence.

### 2.7. Interfacial Transition Assessment and Data Acquisition Strategy

Interfacial behavior was assessed only in the layered groups, Feldspar/ZrO_2_ and Feldspar/Co-Cr, because monolithic ZrO_2_ did not contain a veneer–substrate interface. Interfacial assessment was based primarily on cross-sectional SEM observations, EDS line-scan profiles, and elemental mapping, while XPS provided complementary material-specific surface chemical information. Interfacial transition width was determined from cross-sectional SEM micrographs and normalized EDS line-scan profiles acquired perpendicular to the veneer–substrate interface at an accelerating voltage of 10 kV and a working distance of 10 mm. Line scans were acquired using a step size of 0.20 µm and a probe setting of 3.0, corresponding to an estimated lateral EDS spatial resolution of approximately 0.8–1.0 µm under the selected acquisition conditions.

For each layered specimen, three non-overlapping interfacial locations were selected from morphologically intact cross-sectional regions, avoiding specimen margins, polishing artifacts, sectioning-induced cracks, and visibly contaminated areas. One EDS line scan was acquired at each selected location, resulting in three line scans obtained from spatially distinct locations within each specimen. Background intensity for each elemental signal was determined from compositionally homogeneous regions outside the interfacial transition zone. A signal was considered distinguishable from background when its intensity exceeded the mean background intensity by at least three standard deviations (mean background + 3 SD).

The interfacial transition zone was defined as the full distance interval over which at least one veneer-related elemental signal and at least one substrate-related elemental signal were simultaneously detectable above their respective background thresholds. The transition width was therefore measured between the first and last positions fulfilling this criterion and was not defined by the elemental signal cross-over point. For Feldspar/ZrO_2_, the transition was identified primarily from the spatial overlap between feldspathic ceramic-related signals (Si, Al, Na, and/or K) and zirconia-related signals (Zr and Y). For Feldspar/Co-Cr, the transition was identified from the overlap between feldspathic ceramic-related signals and substrate-related Co and Cr signals, with oxygen interpreted semi-quantitatively. The three transition-width measurements obtained from spatially distinct line scans within each specimen were averaged to generate a single specimen-level value for subsequent statistical analysis.

To improve measurement reproducibility, image acquisition and chemical analyses were performed using a predefined sampling strategy. Fields of view were selected using a systematic sampling approach, avoiding specimen margins, polishing artifacts, cracks caused by sectioning, and visibly contaminated areas. For SEM analysis, five non-overlapping surface fields were acquired from each specimen at 500× magnification. These standardized 500× surface fields served as the sampling units for surface defect density, visible porosity, and microcrack assessment. In the layered groups, three additional cross-sectional fields per specimen were acquired at 2000× magnification and used separately for interfacial evaluation. For EDS analysis, three point spectra were obtained from each predefined region per specimen, including the monolithic zirconia surface, feldspathic veneer, zirconia core, Co-Cr substrate, and interfacial regions, as applicable. Regional point spectra were acquired at 20 kV. Elemental mapping was performed in one representative interfacial area per layered specimen at 10 kV. For XPS analysis, one survey spectrum was acquired from each specimen, resulting in ten XPS acquisitions per experimental group.

For all quantitative analyses, the individual specimen was considered the experimental unit. Multiple SEM fields, EDS point spectra, and EDS line scans acquired within the same specimen were treated as within-specimen subsamples and were not entered into inferential statistical analyses as independent observations. SEM field-level measurements were averaged within each specimen to generate a single specimen-level value for each parameter. Likewise, the three EDS point spectra obtained from each predefined region were averaged to generate one specimen-level regional compositional value, and the three interfacial transition-width measurements obtained from separate line scans were averaged to generate one specimen-level transition-width value. Group-level statistical analyses were therefore based on *n* = 10 independent specimens per experimental group, thereby avoiding pseudoreplication arising from repeated within-specimen measurements.

### 2.8. Statistical Analysis

Statistical analysis was performed according to data type and predefined comparison level. The individual specimen, rather than the individual SEM field, EDS acquisition, or line scan, was used as the unit of statistical analysis. Quantitative SEM-derived parameters, EDS elemental values, interfacial transition width, and XPS atomic percentages, when available, were expressed as mean ± standard deviation. Data distribution was assessed using the Shapiro–Wilk test.

For comparisons involving three experimental groups, normally distributed data were analyzed using one-way analysis of variance followed by Tukey’s post-hoc test, whereas non-normally distributed data were analyzed using the Kruskal–Wallis test followed by Dunn’s post-hoc correction. For predefined two-group comparisons, including interfacial transition width between Feldspar/ZrO_2_ and Feldspar/Co-Cr, Welch’s *t*-test was used for normally distributed data, and the Mann–Whitney U test was used for non-parametric data.

EDS statistical comparisons were restricted to predefined comparable regions and to elements quantitatively detected in both compared regions. Global comparisons across compositionally distinct regions were not performed. Because small numerical differences in elemental composition may reach statistical significance when within-group variability is low, statistically significant EDS differences were interpreted together with their material relevance, regional distribution, and SEM/XPS correlation, rather than as isolated indicators of functional performance. SEM findings were primarily analyzed descriptively, focusing on surface morphology, porosity, microcracks, interfacial continuity, and transition-zone morphology. XPS survey spectra were interpreted qualitatively and semi-quantitatively based on peak identification, relative signal intensity, and atomic percentage values. XPS-derived atomic percentages were expressed as mean ± standard deviation and were compared statistically only for signals quantitatively detected in at least two experimental groups. Because XPS analysis was based primarily on survey spectra and no high-resolution peak deconvolution was performed, these comparisons were interpreted as exploratory semi-quantitative comparisons of surface chemical profiles and were not used for definitive oxide phase identification.

Inter-examiner reliability was assessed from the initial independent evaluations of the two observers. For count-based SEM variables, including surface defect density and microcrack count, agreement was evaluated using the intraclass correlation coefficient (ICC). For ordinal SEM variables, including visible porosity and interfacial continuity scores, agreement was evaluated using weighted Cohen’s kappa (κw). The same reliability measures were used to assess intra-examiner repeatability based on the repeated evaluation of the randomly selected 25% image subset.

The level of statistical significance was set at α = 0.05. Statistical analyses were performed using GraphPad Prism v10.0, and image-based measurements were performed using ImageJ v1.54.

## 3. Results

### 3.1. SEM Morphological Findings

**Figure 1:**
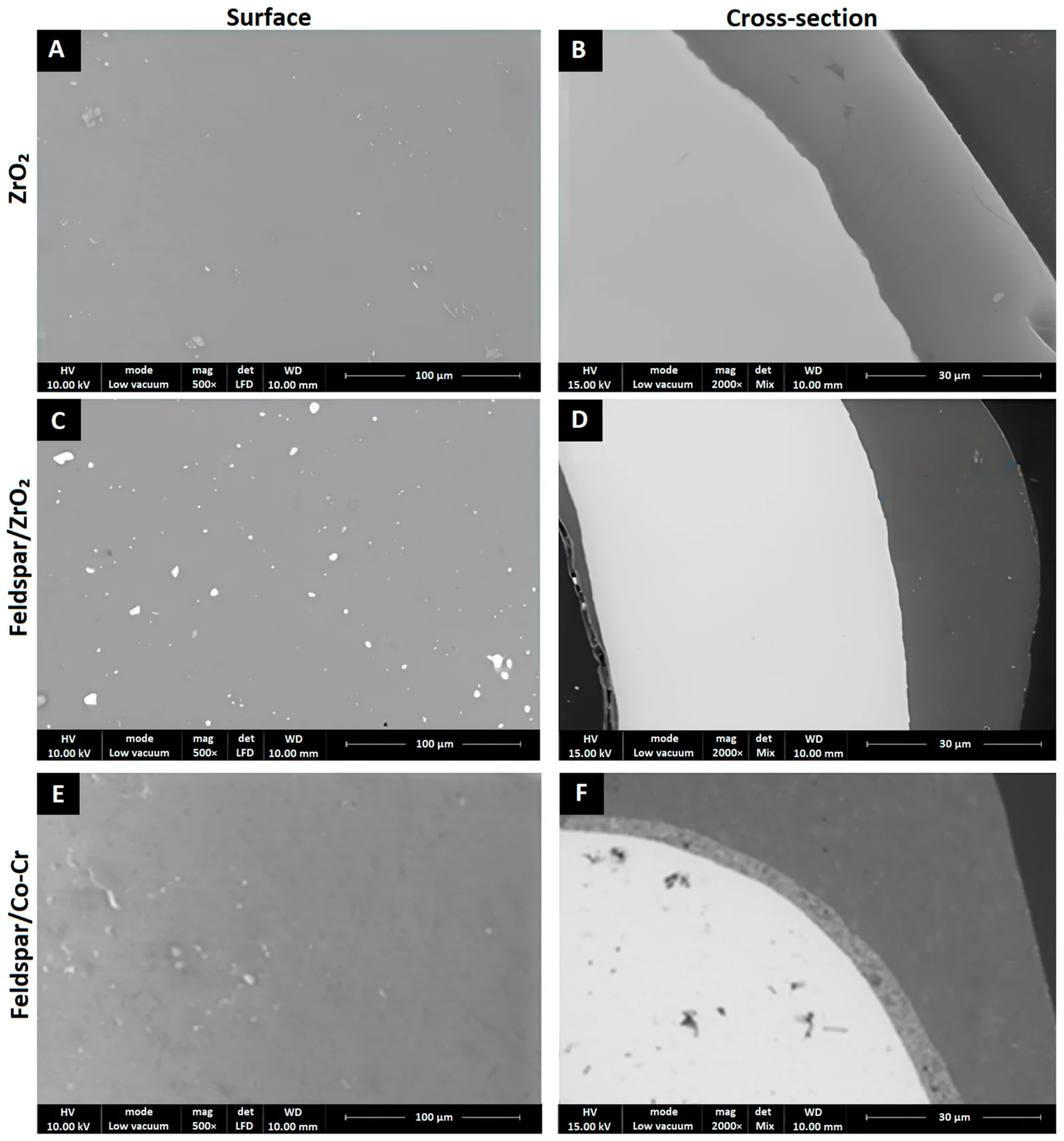
SEM morphological analysis of the analyzed restorative systems. Surface images (**A**,**C**,**E**) were acquired at 500× magnification, whereas cross-sectional images (**B**,**D**,**F**) were acquired at 2000× magnification.

Scanning electron microscopy revealed distinct morphological patterns among the three restorative systems (Figure 1). In the monolithic ZrO_2_ group, surface analysis showed a compact, dense, and relatively homogeneous ceramic structure, without extensive cracks, pores, or structural discontinuities (Figure 1A). Cross-sectional evaluation confirmed the continuity of the zirconia body and the absence of an interfacial region, supporting its role as a reference model for intrinsic monolithic ceramic stability (Figure 1B).

In the Feldspar/ZrO_2_ group, the surface morphology of the feldspathic ceramic appeared continuous, with only localized superficial irregularities (Figure 1C). Cross-sectional analysis showed a clearly identifiable ceramic–ceramic interface between the feldspathic veneer and the zirconia core (Figure 1D). The interfacial boundary appeared continuous, without extensive delamination or gap formation, but the transition between the two ceramic phases was relatively abrupt, suggesting limited interfacial blending.

In the Feldspar/Co-Cr group, the feldspathic ceramic surface showed preserved continuity, with localized micro-irregularities (Figure 1E). Cross-sectional evaluation demonstrated close adaptation of the feldspathic porcelain to the metallic substrate and a more gradual interfacial transition compared with Feldspar/ZrO_2_ (Figure 1F). This pattern was consistent with a broader ceramic–metal interfacial transition region.

The SEM-derived morphometric and semi-quantitative findings are summarized in Table 1. Overall, the three restorative systems showed distinct morphological patterns. Monolithic ZrO_2_ exhibited a compact and homogeneous ceramic morphology with the lowest defect-related values. In the layered systems, interfacial continuity was preserved, with Feldspar/ZrO_2_ showing a sharply defined ceramic–ceramic boundary and Feldspar/Co-Cr showing a broader ceramic–metal transition morphology. No veneer delamination was observed in either layered group.

Inter- and intra-examiner reliability was high for all SEM-derived assessments. Inter-examiner ICC values were 0.94 for surface defect density and 0.92 for microcrack count, while weighted kappa values were 0.88 for visible porosity and 0.90 for interfacial continuity. Across the repeated assessments, intra-examiner reliability coefficients ranged from 0.91 to 0.96 for evaluator 1 and from 0.90 to 0.95 for evaluator 2, indicating consistently high repeatability of the SEM-based assessments.

### 3.2. EDS Elemental Composition

**Figure 2:**
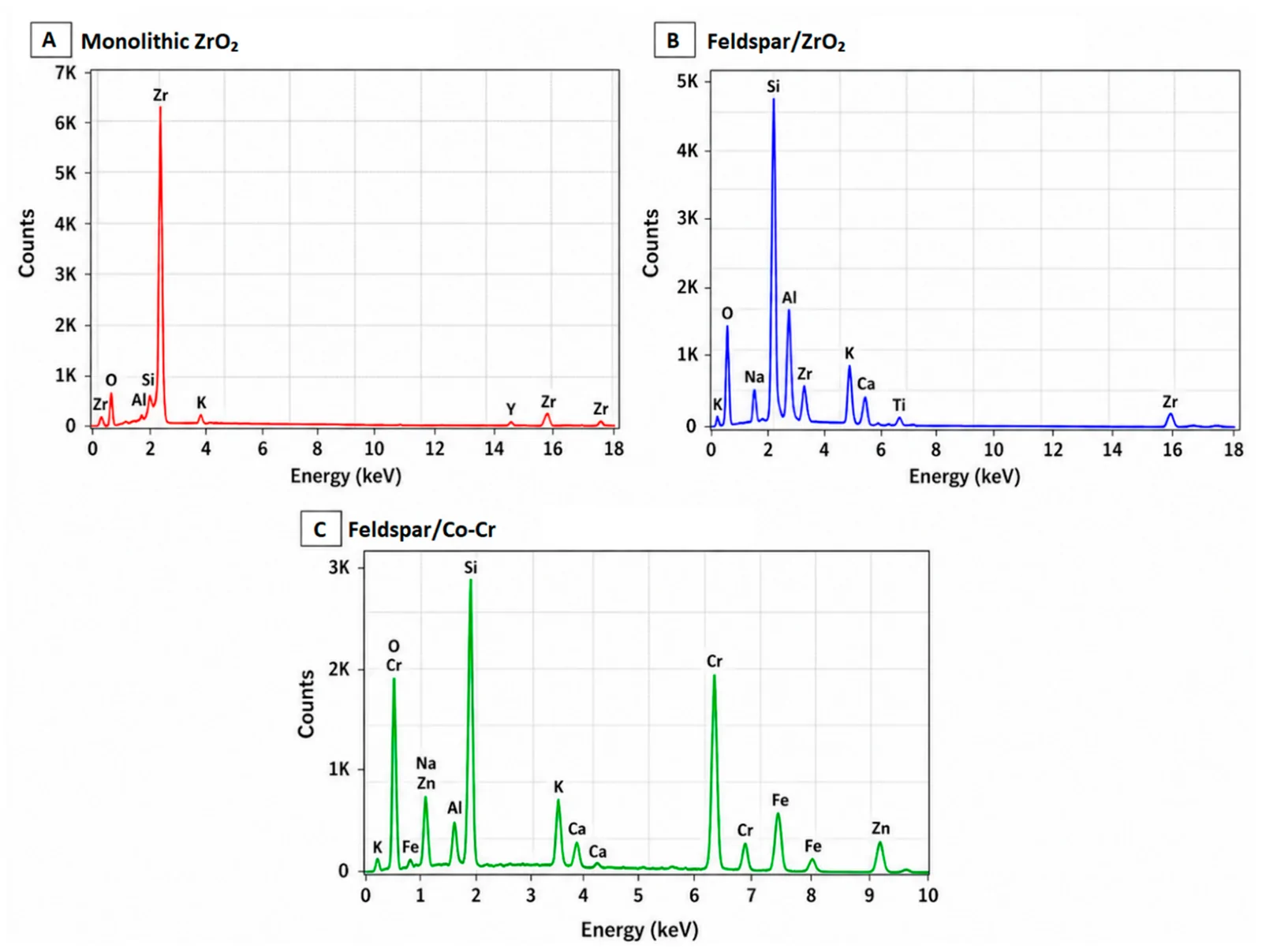
Representative regional EDS point spectra of the analyzed restorative systems acquired at an accelerating voltage of 20 kV: (**A**) monolithic ZrO_2_ bulk/surface region, characterized predominantly by Zr-, O-, and Y-related signals; (**B**) Feldspar/ZrO_2_ representative region, showing feldspathic ceramic-related Si-, O-, Al-, Na-, and K-associated signals together with zirconia-related contributions; and (**C**) Feldspar/Co-Cr representative region, showing ceramic-related signals together with Cr-, Co-, Fe-, and Zn-associated contributions. Spectral intensity is expressed as counts as a function of X-ray energy (keV).

EDS analysis revealed distinct elemental profiles among the three restorative systems (Figure 2). Monolithic ZrO_2_ was characterized mainly by zirconium, oxygen, and yttrium, confirming the expected composition of yttria-stabilized zirconia. Feldspar/ZrO_2_ specimens showed a dual ceramic profile, with silicon, aluminum, sodium, and potassium associated with the feldspathic veneer and zirconium/yttrium associated with the zirconia core. Feldspar/Co-Cr specimens presented feldspathic ceramic-related elements together with cobalt and chromium signals from the metallic substrate. Co-, Cr-, Fe-, and Zn-related signals were mainly identified in the Feldspar/Co-Cr group and were interpreted as group-specific alloy-associated features.

EDS analysis was performed regionally to characterize the elemental composition of the monolithic zirconia surface and the distinct material zones of the layered systems. In Monolithic ZrO_2_, spectra were acquired from the bulk/surface ceramic region, whereas in Feldspar/ZrO_2_ and Feldspar/Co-Cr, spectra were acquired separately from the feldspathic veneer, substrate/core, and interfacial regions. The regional EDS profiles showed a dominant Zr–O–Y composition in Monolithic ZrO_2_ and in the zirconia core of the Feldspar/ZrO_2_ group. Feldspathic veneer regions were characterized mainly by Si, O, Al, Na, and K, while the Co-Cr substrate showed Co- and Cr-dominant signals. The interfacial regions displayed mixed elemental profiles, with feldspathic ceramic-related and Zr/Y-associated signals in Feldspar/ZrO_2_, and ceramic-related together with Co/Cr/O-associated signals in Feldspar/Co-Cr, supporting distinct ceramic–ceramic and ceramic–metal transition behaviors. The regional EDS results are summarized in Table 2.

Statistical analysis of EDS data was restricted to selected interfacial regions and to elements quantitatively detected in both compared systems. Because the analyzed restorative configurations differed in material composition and processing workflow, regional EDS findings were interpreted primarily as descriptive microchemical profiles rather than as global intergroup differences. Direct statistical comparisons between feldspathic veneer regions were not performed because different veneering ceramics were used in the two layered systems. Pairwise statistical testing was therefore limited to selected interfacial EDS parameters and was interpreted as a system-level comparison between the Feldspar/ZrO_2_ and Feldspar/Co-Cr configurations. The results are presented in Table 3.

### 3.3. Interfacial Transition Analysis

**Figure 3:**
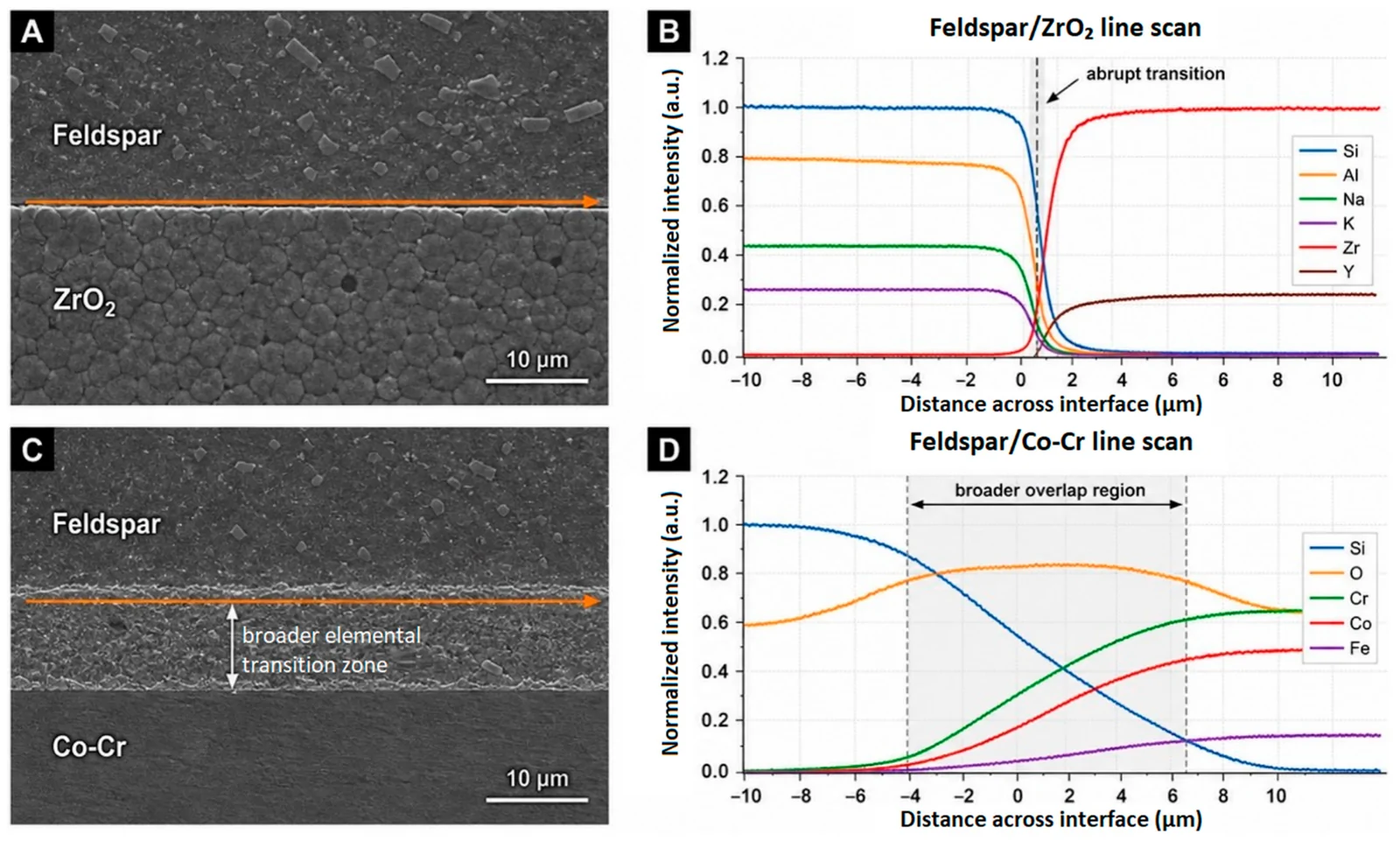
EDS line-scan analysis of the layered restorative systems. Cross-sectional SEM images (**A**,**C**) were acquired at 2000× magnification. Normalized intensity profiles (**B**,**D**) are shown for representative EDS line scans across the veneer–substrate interfaces. Line scans were acquired at an accelerating voltage of 10 kV using a 0.20 µm step size. The interfacial transition width was defined as the full distance interval over which veneer-related and substrate-related elemental signals were simultaneously detectable above background level, rather than as the distance between the signal cross-over points. The orange arrows indicate the EDS line-scan direction across the veneer–substrate interface.

Interfacial transition analysis was performed only for the two layered restorative systems, Feldspar/ZrO_2_ and Feldspar/Co-Cr, because the monolithic ZrO_2_ group did not contain a veneer–substrate interface. Representative SEM-based interfacial regions and corresponding normalized EDS line-scan profiles are presented in Figure 3. The orange arrows indicate the line-scan direction across the veneer–substrate interface. In the Feldspar/ZrO_2_ system, the SEM interfacial image showed the line-scan trajectory across the feldspathic veneer and zirconia core (Figure 3A), while the corresponding elemental profile demonstrated an abrupt transition between feldspathic ceramic-related elements and zirconia-related signals (Figure 3B). In the Feldspar/Co-Cr system, the SEM interfacial image showed the line-scan trajectory across the feldspathic veneer and metallic substrate (Figure 3C), whereas the corresponding elemental profile revealed a broader overlap region mainly involving oxygen, chromium, and cobalt (Figure 3D).

The Feldspar/ZrO_2_ interface showed a narrow and sharply demarcated transition, characterized by a rapid decrease in feldspathic ceramic-related elements and a corresponding increase in zirconia-related signals. In contrast, the Feldspar/Co-Cr interface exhibited a broader elemental overlap region characterized by co-localized O-, Cr-, and Co-related signals. Quantitative comparison confirmed that the interfacial transition width was significantly greater in the Feldspar/Co-Cr group than in the Feldspar/ZrO_2_ group. The statistical comparison, including mean difference, confidence interval, *p* value, and effect size, is summarized in Table 4.

### 3.4. XPS Surface Chemistry

**Figure 4:**
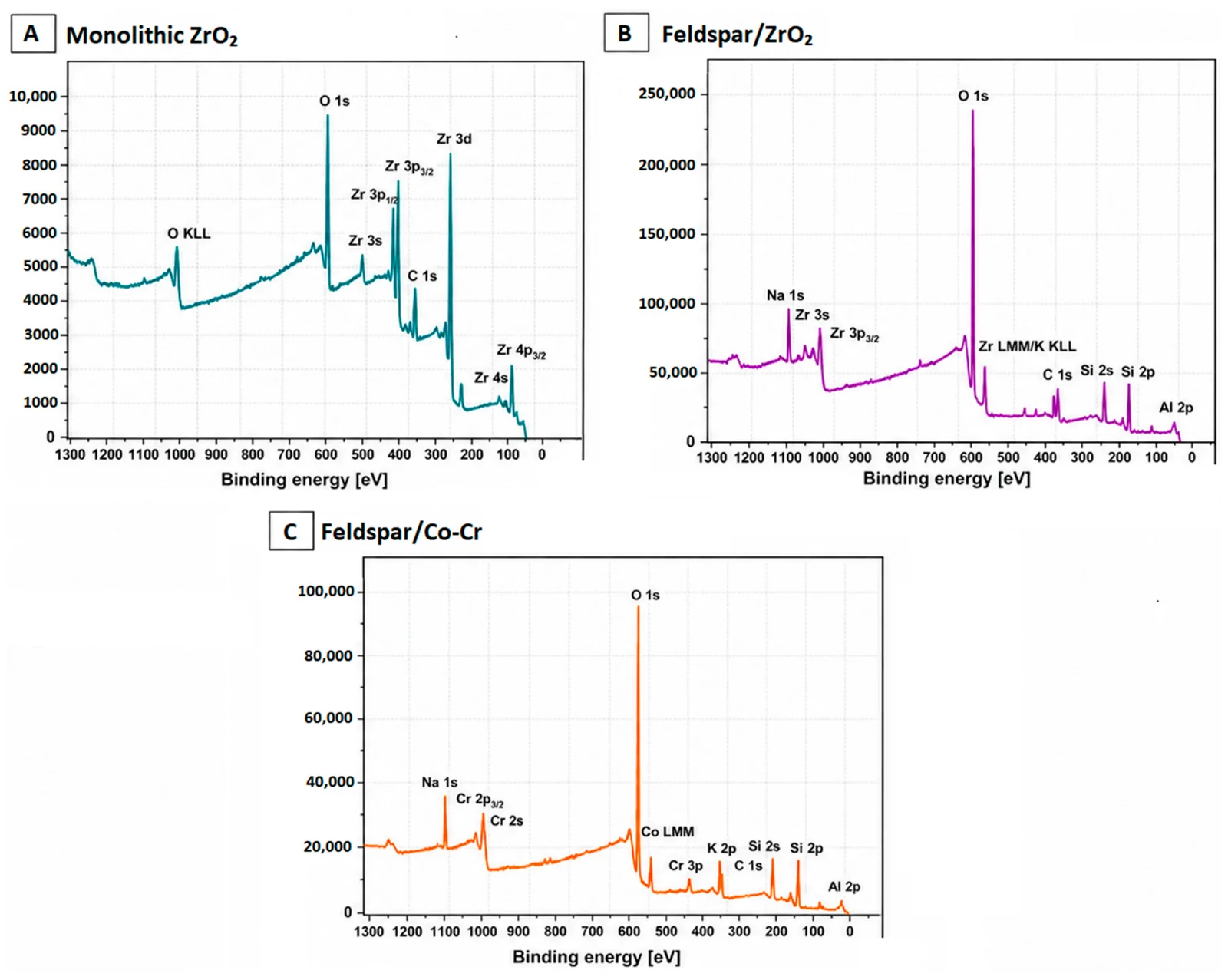
XPS survey spectra of the analyzed restorative systems: (**A**) Monolithic ZrO_2_; (**B**) Feldspar/ZrO_2_; and (**C**) Feldspar/Co-Cr. Spectral intensity is shown as counts as a function of binding energy (eV).

X-ray photoelectron spectroscopy provided complementary information regarding the material-specific surface chemical signatures of the three restorative systems. The XPS survey spectra are presented in Figure 4. Monolithic ZrO_2_ showed zirconium-, oxygen-, and yttrium-related signals, confirming the expected Zr–O–Y surface chemistry of yttria-stabilized zirconia (Figure 4A). This profile supported the oxide-based chemical identity of the zirconia surface under the tested conditions.

In the Feldspar/ZrO_2_ group, the XPS profile was mainly characterized by feldspathic ceramic-related signals, including silicon, oxygen, aluminum, sodium, and potassium, with weak or limited zirconia-associated contributions (Figure 4B). This finding is consistent with the surface sensitivity of XPS and supports the presence of a chemically distinct feldspathic veneering surface. Together with the cross-sectional SEM and EDS findings, this pattern was consistent with a sharply defined ceramic–ceramic interface and limited interfacial chemical intermixing.

In the Feldspar/Co-Cr group, the XPS profile showed feldspathic ceramic-related signals together with limited chromium-, cobalt-, and oxygen-associated contributions (Figure 4C). These findings were interpreted as complementary surface chemical information and were correlated with SEM–EDS observations. Therefore, the broader ceramic–metal elemental transition was mainly supported by cross-sectional SEM morphology and EDS line-scan/mapping data, while XPS provided additional surface chemical context without allowing definitive oxide phase or oxidation-state identification.

The XPS-derived surface chemical composition is summarized in Table 5. The atomic percentage profiles showed distinct surface chemical signatures among the three restorative systems. Monolithic ZrO_2_ presented a dominant Zr–O–Y surface chemistry, whereas Feldspar/ZrO_2_ was mainly characterized by feldspathic ceramic-related Si–O–Al–Na/K signals with weak Zr/Y contributions. Feldspar/Co-Cr showed feldspathic ceramic-related signals together with Cr-, Co-, and O-associated features, supporting an oxide-associated ceramic–metal surface/interfacial contribution without definitive oxide phase assignment. The observed differences in C 1s atomic percentage among groups were attributed to variations in adventitious surface hydrocarbon coverage. These differences may have contributed to partial attenuation of underlying material-specific photoelectron signals, particularly Zr- and Si-related intensities, and were therefore considered when interpreting the XPS atomic percentages as comparative semi-quantitative surface profiles.

Overall, the combined SEM, EDS, and XPS findings demonstrated architecture-dependent differences among the analyzed restorative systems. Monolithic ZrO_2_ showed a compact morphology and stable Zr–O–Y chemistry, Feldspar/ZrO_2_ exhibited a sharply defined ceramic–ceramic interface with limited interfacial intermixing, and Feldspar/Co-Cr presented a broader ceramic–metal elemental transition characterized by Cr-, Co-, and O-related signal overlap, as demonstrated primarily by cross-sectional SEM and EDS line-scan/mapping analysis. These results indicate that restorative architecture was associated with distinct surface chemical profiles and interfacial behaviors.

## 4. Discussion

### 4.1. Principal Findings and Hypothesis Interpretation

The present study demonstrated that the analyzed restorative configurations were associated with distinct microstructural, elemental, surface chemical, and interfacial patterns among the analyzed systems. Monolithic ZrO_2_ showed a compact ceramic morphology and a dominant Zr–O–Y chemical profile, consistent with the expected material-specific morphology and surface chemical characteristics of monolithic zirconia [1,4,8]. Feldspar/ZrO_2_ exhibited a narrow and sharply defined ceramic–ceramic transition, consistent with the interfacial behavior and structural challenges previously described for zirconia–veneering ceramic systems [9,15]. In contrast, Feldspar/Co-Cr showed a broader ceramic–metal transition zone characterized by overlapping Cr-, Co-, and O-related signals. This elemental co-localization is compatible with the known oxidation behavior of Co-Cr metal–ceramic systems; however, the present data do not permit assignment of specific oxidation states or definitive identification of oxide phases. The observed interfacial pattern should therefore be interpreted in the context of the known influence of alloy composition, fabrication route, surface preparation, and thermal processing on metal–ceramic interfaces [16,21]. Importantly, the broader transition zone and elemental overlap observed in the Feldspar/Co-Cr system should not be interpreted as evidence of superior bond strength, mechanical stability, fatigue resistance, or clinical performance, because these functional outcomes were not assessed in the present study.

Accordingly, the principal advance of this study is the quantitative demonstration, under standardized experimental conditions, that the Feldspar/Co-Cr system exhibits a significantly broader interfacial transition than the Feldspar/ZrO_2_ system, together with distinct elemental overlap patterns. The novelty therefore derives from the comparative spatial characterization of the interfaces rather than from confirmation of the expected elemental identities of the constituent materials. These findings describe differences in interfacial microstructural and microchemical organization and do not establish a direct relationship between transition width and functional bonding performance.

These system-level differences should be interpreted at the level of the complete restorative system rather than as effects of architecture alone. Because the experimental groups also differed in substrate composition, veneering ceramic, manufacturing route, and thermal processing, the present design does not permit independent attribution of the observed interfacial differences to any single factor. The measured transition patterns therefore represent the combined outcome of material composition, structural configuration, surface preparation, and fabrication history within each restorative system.

Based on the combined SEM, EDS, and XPS findings, the null hypothesis was rejected. Significant differences were observed among the three restorative systems in terms of surface morphology, elemental composition, surface chemical signatures, and interfacial transition characteristics. These results indicate that Monolithic ZrO_2_, Feldspar/ZrO_2_, and Feldspar/Co-Cr represent distinct restorative configurations characterized by different structural and interfacial patterns: compact monolithic ceramic morphology with a dominant Zr–O–Y chemical profile, a sharply defined ceramic–ceramic interface with limited elemental overlap, and a broader ceramic–metal transition with Cr-, Co-, and O-related elemental overlap, respectively [1,9,14,16,19,20,21]. Because bond-strength, fatigue, aging, and long-term durability tests were not performed, these differences should not be interpreted as evidence of superior bonding, mechanical durability, or clinical performance.

### 4.2. Monolithic ZrO_2_: Compact Morphology and Zr–O–Y Surface Chemical Profile

The Monolithic ZrO_2_ group showed a compact, dense, and relatively homogeneous ceramic morphology, with the lowest defect-related SEM scores among the analyzed systems. This finding is consistent with the structural behavior expected for monolithic zirconia restorations, in which the absence of a veneering ceramic eliminates the veneer–substrate interface and therefore avoids veneer–substrate interface-specific defects such as delamination or veneer chipping [1,2].

The EDS and XPS findings further characterized the material-specific chemical profile of this group, showing a dominant Zr–O–Y composition. This profile reflects the yttria-stabilized zirconia composition and is relevant because zirconia behavior may be influenced by surface chemistry, yttrium-related stabilization, aging, and surface treatment [1,4,5,6,7,8]. However, because no aging, fatigue, or mechanical testing was performed, the present findings should not be interpreted as evidence of superior long-term stability, mechanical performance, or clinical performance.

### 4.3. Feldspar/ZrO_2_: Abrupt Ceramic–Ceramic Interface

The Feldspar/ZrO_2_ group showed a narrow and sharply defined ceramic–ceramic interface, with limited elemental overlap between the feldspathic veneering ceramic and the zirconia core. EDS analysis identified feldspathic ceramic-related elements, mainly Si, O, Al, Na, and K, in the veneering region, whereas Zr and Y were associated with the zirconia substrate, supporting the presence of two chemically distinct ceramic phases [9,10,11,12,13,14]. This pattern is consistent with previous structural and chemical descriptions of zirconia–veneering ceramic interfaces and suggests that interfacial stability in this system is mainly related to ceramic–ceramic adaptation and mechanical continuity rather than extensive chemical intermixing [11,12,13,14,15]. Because zirconia–veneering ceramic bonding is influenced by surface conditioning, veneering technique, liner application, ceramic composition, and interfacial microstructure, this interface remains a critical region in bilayered zirconia restorations, particularly regarding chipping and delamination risks [10,11,12,13,14,15].

### 4.4. Feldspar/Co-Cr: Broader Ceramic–Metal Elemental Transition

The Feldspar/Co-Cr group exhibited a broader interfacial transition zone than the Feldspar/ZrO_2_ group, indicating a more gradual ceramic–metal transition. This finding is consistent with the known influence of Co-Cr alloy composition, manufacturing route, surface preparation, oxidation behavior, and ceramic firing on metal–ceramic interfacial characteristics [16,17,18,19,20,21]. The EDS line-scan/mapping findings demonstrated spatial overlap of Cr-, Co-, and O-related signals within the interfacial region. This elemental co-localization is compatible with the known oxidation behavior of Co-Cr metal–ceramic systems [18,19,20,21]; however, the present data do not permit assignment of specific oxidation states or definitive identification of oxide phases. The XPS analysis was based primarily on survey spectra and did not include high-resolution Cr 2p, Co 2p, or O 1s peak deconvolution. Therefore, specific oxide phases, such as Cr_2_O_3_ or CoO, could not be conclusively identified, and the present results should not be interpreted as definitive evidence of a specific oxide-mediated chemical bonding mechanism. Accordingly, the SEM–EDS findings are interpreted as evidence of a broader ceramic–metal elemental transition characterized by Cr-, Co-, and O-related signal overlap, while XPS provides complementary information on the external surface chemical profile. This interpretation is also consistent with studies showing that Co-Cr processing methods, surface treatments, and thermal cycling can modify microstructure, surface chemistry, ceramic adhesion, and interfacial stability [25,26,27,28].

### 4.5. Integrated SEM–EDS–XPS Interpretation

The combined SEM–EDS–XPS approach provided complementary information on the morphology, elemental distribution, and surface chemistry of the analyzed restorative systems. SEM characterized surface compactness, defect-related features, cross-sectional architecture, and interfacial continuity, while EDS clarified regional elemental distribution and interfacial overlap patterns [14,16,20]. In Feldspar/ZrO_2_, EDS supported a clear separation between feldspathic ceramic-related elements and zirconia-related signals, consistent with a sharply defined ceramic–ceramic interface [14]. In Feldspar/Co-Cr, EDS line-scan/mapping findings supported a broader O–Cr–Co-associated transition, while XPS provided complementary surface chemical information consistent with the material-specific chemical profile without permitting definitive identification of specific oxide species or oxidation states [27,28]. Overall, the integration of SEM, EDS, and XPS showed that monolithic zirconia, ceramic–ceramic layered systems, and metal–ceramic systems differ not only morphologically, but also in microchemical organization and surface chemical behavior [8,16,20].

### 4.6. Material Implications and Interpretation

From a material perspective, the present findings demonstrate that the tested restorative configurations exhibit distinct surface and interfacial microstructural and microchemical patterns under the investigated in vitro conditions. These observations should be interpreted primarily at the material-characterization level and should not be considered direct indicators of clinical performance. Monolithic ZrO_2_ eliminates the veneer–core interface, thereby avoiding veneer–core interface-specific structural features [1,2]. However, because no aging, fatigue, or mechanical testing was performed, the present study does not provide evidence regarding its long-term mechanical stability or clinical durability.

In Feldspar/ZrO_2_ restorations, the sharply defined ceramic–ceramic interface indicates limited elemental overlap between the veneering ceramic and zirconia core under the present experimental conditions. Previous studies have shown that veneering protocol, liner application, surface treatment, and interfacial characteristics can influence zirconia–veneer bonding behavior [9,10,11,12,13,14,15]. The present findings, however, characterize interfacial morphology and elemental distribution only and do not establish the mechanical strength or durability of this interface.

In Feldspar/Co-Cr restorations, the broader ceramic–metal transition is consistent with the recognized influence of alloy composition, manufacturing route, surface preparation, oxidation behavior, and ceramic firing on metal–ceramic interfacial characteristics [18,19,20,21]. Nevertheless, the broader transition zone and Cr-, Co-, and O-related elemental overlap observed in this study should not be interpreted as evidence of superior ceramic–metal bonding, fatigue resistance, mechanical stability, or clinical performance.

Accordingly, the present results highlight material-specific differences in interfacial organization and surface chemistry rather than differences in functional performance. Their clinical significance remains to be established through studies incorporating bond-strength testing, fatigue loading, thermocycling, aging protocols, and long-term mechanical evaluation.

### 4.7. Limitations and Future Research

The present study has several limitations. First, the investigation was conducted under in vitro conditions; therefore, the findings cannot be directly extrapolated to clinical performance. Second, although the sample size was adequate for comparative microstructural assessment, it remains relatively limited. An additional limitation is that restorative architecture was not experimentally isolated from material composition and processing variables. The compared systems differed in substrate material, veneering ceramic, manufacturing route, oxidation treatment, and firing protocol. Consequently, the observed differences cannot be attributed exclusively to restorative architecture and should instead be interpreted as system-level differences among the tested restorative configurations. Third, the specimens were not subjected to thermocycling, mechanical aging, fatigue loading, or long-term hydrothermal degradation, which may influence interfacial behavior under clinical conditions. Fourth, no shear bond strength or fracture resistance tests were performed; therefore, the observed interfacial transition width and elemental overlap patterns should not be interpreted as indicators of superior bonding, fatigue resistance, long-term mechanical stability, or clinical performance. Finally, XPS analysis was based mainly on survey spectra, without high-resolution peak deconvolution; consequently, Cr-, Co-, and O-related surface chemical features were interpreted cautiously and were not used for definitive oxide phase identification or assignment of specific oxidation states.

Future studies should combine SEM–EDS–XPS characterization with artificial aging protocols and mechanical testing, particularly shear bond strength and fracture resistance assessments, to determine whether interfacial transition width and elemental overlap patterns are associated with functional performance. High-resolution XPS peak deconvolution should also be used to clarify oxide-related chemical states at the ceramic–metal interface. Further investigations should compare the influence of different Co-Cr manufacturing routes, including casting, milling, and selective laser melting, on interfacial morphology, surface chemical characteristics, and ceramic–metal bonding behavior [18,20,26,27]. Future factorial studies using controlled combinations of substrate, veneering ceramic, and fabrication workflow will be required to determine the independent contribution of each variable.

## 5. Conclusions

Within the limitations of this in vitro study, the analyzed restorative configurations exhibited distinct morphological, elemental, and surface chemical behaviors. Monolithic ZrO_2_ showed a compact ceramic morphology and a dominant Zr–O–Y surface profile, consistent with its material-specific microstructural and surface chemical characteristics under the tested conditions. Feldspar/ZrO_2_ exhibited a sharply defined ceramic–ceramic interface with limited elemental overlap, whereas Feldspar/Co-Cr showed a broader ceramic–metal transition, mainly supported by SEM–EDS evidence of Cr-, Co-, and O-related interfacial signals. These findings indicate that monolithic zirconia, zirconia-based bilayered restorations, and Co-Cr-based metal–ceramic systems present distinct interfacial and microchemical patterns. However, because bond-strength testing, fatigue loading, thermocycling, and aging protocols were not performed, the observed transition width and elemental overlap patterns should not be interpreted as evidence of superior bonding, mechanical stability, durability, or clinical performance. Integrated SEM–EDS–XPS assessment may therefore provide useful complementary information for understanding system-dependent surface chemistry and interfacial behavior in dental restorative materials, while the functional significance of these differences requires confirmation through mechanical and aging studies.

## Figures and Tables

**Table 1:** SEM-derived morphometric and semi-quantitative parameters of the analyzed restorative systems.

SEM Parameter	Monolithic ZrO_2_	Feldspar/ZrO_2_	Feldspar/Co-Cr	*p* Value
Surface defect density, defects/field	1.2 ± 0.4	2.1 ± 0.6	2.6 ± 0.8	<0.05
Visible porosity score	0.3 ± 0.2	0.6 ± 0.3	0.9 ± 0.4	<0.05
Microcrack count, cracks/field	0.2 ± 0.1	0.5 ± 0.2	0.6 ± 0.3	<0.05
Interfacial continuity score	NA	3.8 ± 0.3	3.5 ± 0.4	0.08 *
Veneer delamination incidence, *n*/N	NA	0/10	0/10	NA
Interface presence	NA	Present	Present	NA

Note: Quantitative values are presented as mean ± standard deviation (SD), unless otherwise specified. NA = not applicable. Surface defect density and microcrack count were determined from five standardized non-overlapping surface SEM fields acquired at 500× magnification for each specimen and are expressed as counts per field. Visible porosity was assessed on the same standardized surface fields using a four-point semi-quantitative score, where 0 indicated absent/negligible porosity and 3 indicated marked porosity. The mean of the five field-level observations was calculated for each specimen, and specimen-level values were used for group-level statistical analysis. Interfacial continuity was assessed only in the layered groups using a five-point score, where 1 indicated marked discontinuity/gap formation and 5 indicated a continuous interface without visible gaps or delamination. Veneer delamination incidence is reported as the number of affected specimens/total specimens. * The *p* value for interfacial continuity score refers only to the comparison between Feldspar/ZrO_2_ and Feldspar/Co-Cr.

**Table 2:** Regional EDS elemental composition of monolithic and layered restorative systems.

Element/ Indicator	MZ Bulk/ Surface	FZ Veneer	FZ Core	FZ Interface	FC Veneer	FC Substrate	FC Interface
O, wt%	25.80 ± 1.00	48.10 ± 0.50	32.40 ± 1.10	40.80 ± 1.20	48.60 ± 0.70	2.80 ± 0.60	38.50 ± 1.40
Na, wt%	<0.1	7.30 ± 0.20	<0.1	3.20 ± 0.30	6.80 ± 0.30	ND	3.60 ± 0.40
Mg, wt%	ND	ND/trace	ND	ND	ND/trace	ND/trace	<0.1
Al, wt%	0.05 ± 0.02	12.40 ± 0.20	0.05 ± 0.02	5.80 ± 0.50	9.50 ± 0.40	ND/trace	4.10 ± 0.50
Si, wt%	1.50 ± 0.20	26.70 ± 0.30	1.20 ± 0.20	13.80 ± 0.80	27.40 ± 0.60	0.80 ± 0.20	15.80 ± 0.90
K, wt%	0.40 ± 0.06	5.50 ± 0.20	0.40 ± 0.07	2.60 ± 0.30	4.70 ± 0.30	ND	2.40 ± 0.30
Ca, wt%	<0.1	ND/trace	<0.1	<0.1	1.20 ± 0.20	ND	0.70 ± 0.20
Ti, wt%	<0.1	ND	<0.1	ND	ND	ND	ND
Zn, wt%	<0.1	ND	<0.1	ND/trace	ND/trace	ND/trace	2.50 ± 0.40
Y, wt%	2.40 ± 0.30	ND/trace	6.10 ± 0.40	2.80 ± 0.30	ND	ND	ND
Zr, wt%	69.70 ± 1.20	ND/trace	59.80 ± 1.30	30.90 ± 1.10	ND	ND	ND
Cr, wt%	ND	ND	ND	ND	ND/trace	28.00 ± 1.20	13.20 ± 0.90
Fe, wt%	ND	ND	ND	ND	ND/trace	1.50 ± 0.40	1.90 ± 0.40
Co, wt%	ND	ND	ND	ND	ND/trace	66.90 ± 1.50	17.30 ± 1.10
Mo/W, wt%	ND	ND	ND	ND	ND	5.00 ± 0.80	2.00 ± 0.50

Abbreviations: MZ = Monolithic ZrO_2_; FZ = Feldspar/ZrO_2_; FC = Feldspar/Co-Cr; V = veneer; C = core; S = substrate; I = interface; ND = not detected. Note: Quantitative values are presented as mean ± standard deviation (SD) and reported as weight percentages (wt%), unless otherwise specified. ND = not detected; trace = element detected close to the analytical detection limit. For layered systems, EDS values are reported by analyzed region, including feldspathic veneer, substrate/core, and interface. The dominant chemical signatures summarize the principal elemental profile of each analyzed region and were used for descriptive interpretation together with SEM and XPS findings. Because these regions represent compositionally distinct material zones, global *p* values were not calculated for this table. Oxygen wt% values should be interpreted semi-quantitatively because EDS-based light-element quantification may be influenced by matrix effects and acquisition conditions. Mo- and W-related contributions were reported jointly because their individual quantitative separation was considered less reliable; these contributions were therefore interpreted semi-quantitatively.

**Table 3:** Statistical comparison of selected comparable EDS regions and interfacial transition width.

Comparison	Element/Parameter	Group 1	Group 2	*p* Value
FZ-I vs. FC-I	Si, wt%	13.80 ± 0.80	15.80 ± 0.90	<0.001
FZ-I vs. FC-I	Al, wt%	5.80 ± 0.50	4.10 ± 0.50	<0.001
FZ-I vs. FC-I	Na, wt%	3.20 ± 0.30	3.60 ± 0.40	0.023
FZ-I vs. FC-I	K, wt%	2.60 ± 0.30	2.40 ± 0.30	0.154
FZ-I vs. FC-I	O, wt%	40.80 ± 1.20	38.50 ± 1.40	0.001

Abbreviations: FZ-I = Feldspar/ZrO_2_ interface; FC-I = Feldspar/Co-Cr interface. Note: Quantitative values are presented as mean ± standard deviation (SD). Pairwise comparisons were performed using Welch’s *t*-test. Because different veneering ceramics were used in the two layered restorative systems, direct statistical comparisons between feldspathic veneer regions were not performed. Statistical testing was restricted to selected interfacial EDS parameters and was interpreted as a system-level comparison between the two layered restorative configurations rather than as an isolated effect of substrate composition or restorative architecture. Comparisons involving oxygen were interpreted with caution because EDS-based oxygen quantification is semi-quantitative.

**Table 4:** Statistical comparison of interfacial transition width between layered restorative systems.

Parameter	Feldspar/ ZrO_2_	Feldspar/ Co-Cr	Mean Difference	95% CI for Mean Difference	Welch’s *t*-Test	*p* Value	Effect Size
Interfacial transition width, µm	1.7 ± 0.4	3.8 ± 0.6	2.10	1.62–2.58	t = 9.21; df = 15.68	<0.001	Cohen’s d = 4.12

Note: Quantitative values are presented as mean ± standard deviation (SD). The mean difference was calculated as Feldspar/Co-Cr minus Feldspar/ZrO_2_. CI = confidence interval. The monolithic ZrO_2_ group was not included because no veneer–substrate interface was present.

**Table 5:** XPS-derived surface chemical composition of the analyzed restorative systems.

XPS Signal/Indicator	Monolithic ZrO_2_, at%	Feldspar/ZrO_2_, at%	Feldspar/Co-Cr, at%	*p* Value
O 1s	58.52 ± 1.16	53.79 ± 1.06	50.42 ± 1.38	<0.001
Zr-related signals	24.26 ± 1.26	3.05 ± 0.54	ND	<0.001 *
Y-related signals	2.88 ± 0.28	0.40 ± 0.18	ND	<0.001 *
Si-related signals	0.79 ± 0.41	22.72 ± 0.89	20.19 ± 0.91	<0.001
Al-related signals	0.30 ± 0.09	6.88 ± 0.72	7.13 ± 0.58	<0.001
Na/K-related signals	0.17 ± 0.10	5.37 ± 0.49	5.19 ± 0.51	<0.001
Cr-related signals	ND	ND	4.83 ± 0.39	NA
Co-related signals	ND	ND	3.78 ± 0.54	NA
C 1s	13.23 ± 0.96	6.53 ± 0.63	8.91 ± 1.39	<0.001

Note: Quantitative values are presented as mean ± standard deviation (SD) and reported as atomic percentages (at%), unless otherwise specified. ND = not detected; NA = not applicable. *p* values refer only to exploratory intergroup comparisons performed for XPS signals quantitatively detected in at least two experimental groups. * For Zr- and Y-related signals, statistical comparison was performed only between zirconia-containing groups. XPS findings were interpreted as comparative semi-quantitative surface chemical profiles and were not used for definitive oxide phase identification in the absence of high-resolution peak deconvolution.

## Data Availability

The original contributions presented in this study are included in the article. Further inquiries can be directed to the corresponding authors.

## Abbreviations

The following abbreviations are used in this manuscript:

Al	Aluminum
Al Kα	Aluminum K-alpha radiation
at%	Atomic percentage
CAD/CAM	Computer-aided design/computer-aided manufacturing
C 1s	Carbon 1s photoelectron signal
Ca	Calcium
CI	Confidence interval
Co	Cobalt
Co-Cr	Cobalt–chromium alloy
Cr	Chromium
df	Degrees of freedom
EDS	Energy-dispersive X-ray spectroscopy
FC	Feldspar/Co-Cr
FC-I	Feldspar/Co-Cr interface
FC-S	Feldspar/Co-Cr substrate
FC-V	Feldspar/Co-Cr feldspathic veneer
Fe	Iron
FZ	Feldspar/ZrO_2_
FZ-C	Feldspar/ZrO_2_ core
FZ-I	Feldspar/ZrO_2_ interface
FZ-V	Feldspar/ZrO_2_ feldspathic veneer
K	Potassium
Mg	Magnesium
Mo	Molybdenum
MZ	Monolithic ZrO_2_
NA	Not applicable
Na	Sodium
ND	Not detected
O	Oxygen
O 1s	Oxygen 1s photoelectron signal
SEM	Scanning electron microscopy
Si	Silicon
Ti	Titanium
W	Tungsten
wt%	Weight percentage
XPS	X-ray photoelectron spectroscopy
Y	Yttrium
Y-TZP	Yttria-stabilized tetragonal zirconia polycrystal
Zn	Zinc
Zr	Zirconium
ZrO_2_	Zirconia/zirconium dioxide
µm	Micrometer

## References

[1] Cesar P.F., Miranda R.B.P., Santos K.F., Scherrer S.S., Zhang Y. (2024). Recent advances in dental zirconia: 15 years of material and processing evolution. Dent. Mater..

[2] Cuzic C., Rominu M., Pricop A., Urechescu H., Pricop M.O., Rotar R., Cuzic O.S., Sinescu C., Jivanescu A. (2025). Clinician’s Guide to Material Selection for All-Ceramics in Modern Digital Dentistry. Materials.

[3] Almulhim K.S., Syed M.R., Alqahtani N., Alamoudi M., Khan M., Ahmed S.Z., Khan A.S. (2022). Bioactive Inorganic Materials for Dental Applications: A Narrative Review. Materials.

[4] Kim S.-H., Oh K.C., Moon H.-S. (2024). Effects of Surface-Etching Systems on the Shear Bond Strength of Dual-Polymerized Resin Cement and Zirconia. Materials.

[5] Conner C., Andretti F., Hernandez A.I., Rojas-Rueda S., Azpiazu-Flores F.X., Morrow B.R., Garcia-Godoy F., Jurado C.A., Alshabib A. (2025). Surface Evaluation of a Novel Acid-Etching Solution for Zirconia and Lithium Disilicate. Materials.

[6] Alkhudhairy F., Shono N.N. (2025). An investigation of Y-TZP surface characteristics and adhesive properties following treatment with Tri-biochemical Silica Coating, Femtosecond Laser, and Nano-hydroxyapatite: A scanning electron microscopy evaluation. Pak. J. Med. Sci..

[7] Pandoleon P., Kontonasaki E., Kantiranis N., Pliatsikas N., Patsalas P., Papadopoulou L., Zorba T., Paraskevopoulos K.M., Koidis P. (2017). Aging of 3Y-TZP dental zirconia and yttrium depletion. Dent. Mater..

[8] Valverde G.B., Coelho P.G., Janal M.N., Lorenzoni F.C., Carvalho R.M., Thompson V.P., Weltemann K.D., Silva N.R. (2013). Surface characterisation and bonding of Y-TZP following non-thermal plasma treatment. J. Dent..

[9] Zicari F., Monaco C., Vivan Cardoso M., Silvestri D., Van Meerbeek B. (2024). Bonding Effectiveness of Veneering Ceramic to Zirconia after Different Grit-Blasting Treatments. Dent. J..

[10] AlHelal A.A., Habib S.R., Alyousef S., Alhamzah N., Alqahtani M., Alqahtani A. (2025). Bond strength between layering ceramic and zirconia core: Influence of various surface conditioning of zirconia. PeerJ.

[11] Dimitriadis K., Tulyaganov D.U., Agathopoulos S. (2024). Evaluation of bond strength between zirconia milled ceramic material and veneered dental porcelain. Eur. J. Oral Sci..

[12] Nogueira V.F., Rodrigues C.D.S., Grangeiro M.T.V., Contreras L.P.C., Marinho R.M.M., Bottino M.A. (2024). Interface adhesion on layered zirconia: Effects of the veneering ceramic material and veneering technique. J. Prosthodont..

[13] Youssef M.K., Abdelkader S.H., Aly Y.M. (2023). Effect of different interfacial surface treatments on the shear bond strength of veneering ceramic and zirconia core. BMC Oral Health.

[14] Inokoshi M., Yoshihara K., Nagaoka N., Nakanishi M., De Munck J., Minakuchi S., Vanmeensel K., Zhang F., Yoshida Y., Vleugels J. (2016). Structural and Chemical Analysis of the Zirconia-Veneering Ceramic Interface. J. Dent. Res..

[15] Maroiu A.C., Luca M.M., Jivanescu A. (2026). Effect of Lithium-Disilicate Liners on Bond Strength and Fracture Resistance of Bilayered Zirconia Systems: A Systematic Review of In Vitro Evidence. Dent. J..

[16] Dawod N., Miculescu M., Antoniac I.V., Miculescu F., Agop-Forna D. (2023). Metal-Ceramic Compatibility in Dental Restorations According to the Metallic Component Manufacturing Procedure. Materials.

[17] Kassapidou M., Stenport V.F., Johansson C.B., Syverud M., Hammarström Johansson P., Börjesson J., Hjalmarsson L. (2023). Cobalt chromium alloys in fixed prosthodontics: Investigations of mechanical properties and microstructure. J. Prosthet. Dent..

[18] Daou E.E., Özcan M. (2025). Comparison of Ceramic Bonding to Cobalt-Chromium, Zirconia and Nickel-Chromium Alloys Fabricated Using of Various Techniques. J. Biomed. Mater. Res. B Appl. Biomater..

[19] Hong J.K., Kim S.K., Heo S.J., Koak J.Y. (2020). Mechanical Properties and Metal-Ceramic Bond Strength of Co-Cr Alloy Manufactured by Selective Laser Melting. Materials.

[20] Revilla-León M., Al-Haj Husain N., Methani M.M., Özcan M. (2021). Chemical composition, surface roughness, and ceramic bond strength of additively manufactured cobalt-chromium dental alloys. J. Prosthet. Dent..

[21] Li J., Chen C., Liao J., Liu L., Ye X., Lin S., Ye J. (2017). Bond strengths of porcelain to cobalt-chromium alloys made by casting, milling, and selective laser melting. J. Prosthet. Dent..

[22] Park W.U., Park H.G., Hwang K.H., Zhao J., Lee J.K. (2017). Interfacial Property of Dental Cobalt–Chromium Alloys and Their Bonding Strength with Porcelains. J. Nanosci. Nanotechnol..

[23] Stoian C., Ardelean L.C., Negrutiu M.L., Miclau M., Casut C., Sinescu C., Alexandratou A., Katsavrias A., Stoian A.D., Rominu M. (2023). Crystalline Structure Assessment of Ceramic Veneered Co-Cr-W Dental Alloy Substructures Obtained by Selective Laser Melting—A Pilot Study. Appl. Sci..

[24] Wojciechowski I., Klatkiewicz T., Prylińska-Czyżewska A., Borczyńska A., Jakubowska W., Pryliński M. (2024). Methods of Processing Dental Chromium-Cobalt Alloys for Production of Metal Frameworks Faced with Ceramics to Obtain the Best Mechanical Properties. Med. Sci. Monit. Basic Res..

[25] Daou E.E., Özcan M., Salameh P., Salameh Z. (2024). Effect of Ceramic Veneering on the Microstructure of Pre-sintered Cobalt-Chromium, Compared to Pre-sintered Zirconia and Conventional Cast Alloys. Front Dent..

[26] Pruszczyńska E., Kula Z., Dąbrowska K., Klimek L. (2025). The Shear Bond Strength of Porcelain Bonding to Cobalt-Chromium Dental Alloys Before and After Thermal Cycling. Metals.

[27] Uriciuc W.-A., Suciu M., Barbu-Tudoran L., Botean A.-I., Chicinaș H.F., Anghel M.-A., Popa C.O., Ilea A. (2025). Surface Treatments on Cobalt–Chromium Alloys for Layering Ceramic Paint Coatings in Dental Prosthetics. Coatings.

[28] Ziębowicz A., Pawlyta M. (2025). Atomic Layer Deposition of Zirconia on Cobalt–Chromium Alloys for Dental Prosthetics: Surface Functionalization Under MDR 2017/745. Coatings.

